# Patient safety and surgical innovation–complementary or mutually exclusive?

**DOI:** 10.1186/1754-9493-8-17

**Published:** 2014-04-02

**Authors:** Dan E Azagury

**Affiliations:** 1 Minimally Invasive & Bariatric Surgery, Stanford University School of Medicine, Stanford, USA

## Safety vs. innovation

Safety and innovation are not and need not be antagonists, but synonyms. If innovation is driven by safety, we can hopefully accelerate the rate of innovation desperately needed in medicine.

In the surgical microcosm, it is not infrequent to hear the voices of surgical conservatism question innovation: why change the current device or method? It is proven to work, it is vetted by years of clinical research, and should therefore be chosen–because it’s safer. There’s nothing wrong with that thought process, is there?

There is no doubt that safety is the cornerstone of our practice. However, caution and prudence should not be deterrents for innovation. On the contrary, if younger surgeons or trainees have the luxury of being attracted to innovation, it is only because their mentors use established methods and strategies. This creates a setting of safety and security in which younger surgeons are rooted. The established surgical practice needs to be one of absolute focus on safety; only then can the younger generation be comfortable enough with the current technologies to *look into its shortcomings*.

Just like creating a safe household and offering children a rigid set of ground rules and education, anchoring residents in safety is a critical initial step. But at some point, your children will need to experience their own life. And if you hamper that, your kids will never surpass you… The role of mentors is therefore to teach and give trainees the tools to practice surgery safely. This will allow them to recreate the same environment of absolute attention to safety for their own patients. Surgical curriculums are aimed at training residents to reach this stage, not to surpass it. It is good, but not good enough. Once surgical training has been appropriately achieved, it is also the role of mentors to push trainees to venture out further and not fall asleep in the safe mode. They should be encouraged to keep searching. If we want our field to evolve, we owe it to ourselves, to our patients, and to our trainees not to stop at simply reproducing what we have learned, but to improve it.

It is thus our role, as teachers, to encourage a *mindset* of innovation in medical students and residents, and give them the tools to pursue innovation safely.

## Finding unmet needs in surgical practice?

Innovation and surgery can be two facets to the same person, the same career. However, since the times of illustrious surgeon/inventors who could bring their inventions directly from the lab to the operating room, things have changed. Just like surgeons pursuing basic research don’t inject the stem cells they grow in their lab in the patient they care for on the floor, clinical practice is not a place to carelessly experience with innovation. And current rules and regulations ensure that fact thoroughly. However, clinical practice is a unique set for identification of unmeet clinical needs. The surgeon has the unique “privilege” of witnessing the shortcomings of current treatments and their impact on patients. It is her or his duty to use this “privilege” to improve care. The best innovation, and the safest innovation, is the one grounded in real problem solving needs. *Only if we identify problems worth solving, will we develop solutions worth pursuing.* And as surgeons, these solutions often take the shape of devices, which is exactly what medtech innovation is: Medical or surgical technology (Medtech) innovation refers the process whereby scientific discoveries, which could solve clinical problems, are driven forward across the translational gap into a device used in clinical practice [1,2].

## Training to innovate

So you’ve identified an unmet need. Maybe invented a solution… Now what?

Navigating the requirement of medical technology development is actually only getting more and more complex: what regulatory pathway is required in the US for the device to be FDA cleared or approved, or CE marked in Europe? What and how to patent your invention? What will be required to demonstrate efficacy? What are the possible engineering challenges and how can they be averted? All these aspects need to be taken into account early on in the process of innovation. If only because funding such an endeavor is already difficult enough that investors will not finance promising devices if they see multiple major barriers in the development road ahead.

**Figure 1:**
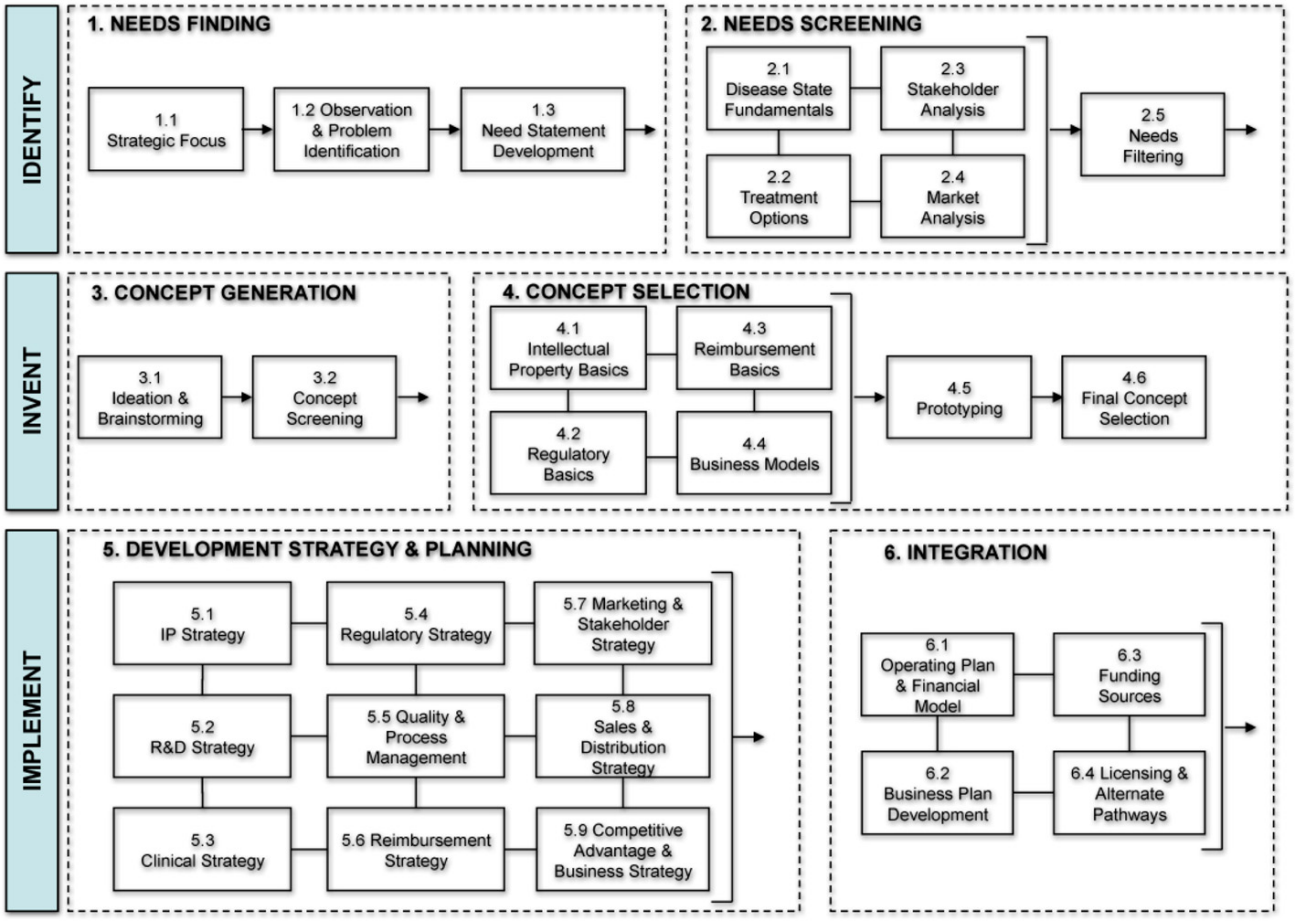
The Stanford Biodesign Process (© Stanford Biodesign, used with permission).

Theses aspects of innovation are not part of our core training, but programs exist however to fill that gap and teach innovation. Just like the rest of surgery, there is a process, and it can be learned and taught. These programs are flourishing across universities around the world: The Stanford Biodesign Program, was created for that purpose 12 years ago to teach physicians and engineers the process of medtech innovation (Figure 1) [3,4].

It is currently available in multiple formats, including a one-year full time fellowship: this is a team-based approach (2-3 engineers, and 1 or 2 physicians) combining theoretical knowledge and practical experience. Courses include intellectual property, regulatory pathway, market overview, funding sources and mechanisms, etc. Then, teams are pared up with a medical department in the adjacent hospital in order to directly observe patient care and identify unmet clinical needs. The teams will then follow the entire process of identification, invention and implementation of the concept they have selected and will often keep pursuing their innovation after the fellowship is over.

Innovation programs such as the one from Stanford University have flourished across the United States and Europe, with sometimes different focuses such as Image Guided Surgery innovation or Radiology innovation, etc. and are currently grouped into BME-IDEA: the Biomedical Engineering–Innovation, Design and Entrepreneurship Alliance (http://www.stanford.edu/group/biodesign/cgi-bin/bme-idea/).

## Urgent call for innovation

In a time of widespread technophilia in our daily life, lack of innovation in medical technologies is actually not uncommon. It is difficult to quantify the amount of innovation in our field. However, it still strikes me than medical technologies are lagging behind compared to the revolution outside the hospital. Our daily life is all about technology: every place, every time, for everyone. And I might be wrong, but as far as innovation in our operating room is concerned, I can’t seem to have the same feeling of living a period of intense and extremely rapid evolution. You can argue that laparoscopy or endoscopy or minimally invasive technologies revolutionized surgery, but at what pace?

Let’s take an example. Laparoscopy. Most of us would agree that laparoscopy has been a revolution in surgery. But Dr. Jacobaeus performed the first laparoscopy in 1911! Since then we have invented the radio, the television and computers (and we went to the moon). The first laparoscopic surgery was performed in 1980. Since then we’ve invented digital cell phones, personal computers, the internet and self driving electrical cars… Not exactly a comparable leap forward.

Another example? Surgical staplers have allowed advanced laparoscopic procedures but what is the pace of that invention: “Some doctors from the empirical sect have reported this mode of treatment for small intestinal wounds. We take large headed ants, bring together the wound edges and apply the and with gaping mouth to both edges. Once the ant closes her mouth, cut off its head which then stays adherent and doesn’t fall off. We then take another ant and place it near the first one and we repeat the operation until the entire length of the wound is closed. The intestines are then returned into the abdomen and the wound is ligated. Now the heads stay adherent to the intestines until it heals without any complication to the patient.” Kahlef ben Abbâs Aboulcassem Ezzaharaoui–Aka–Abulcasis in “Al-Tasrif” ca. 1000 AD.

So even if this very subjective comparison is inherently biased, it is difficult to justify that the rate of surgical innovation has been mind-blowing by any standards. There has been some remarkable innovation in surgery over the past century, but I am sure we can do better.

## Take-home message

We owe ourselves more and better innovation in our surgical fields. Safety and innovation are not antinomies, and we can thrive for innovation without ever compromising on safety. Safely innovating means bringing innovation outside of the operating room in an innovation structure: protecting our patients by creating an innovation specialty that can be practiced in innovation labs and taught in innovation programs. We have to go beyond our comfort zone of reproducing safe procedures to the uncharted area of needs finding and innovation. And while innovating is a risky process, risk may only lie on the innovator, not the patient. So while it is our role as surgeons and doctors to practice safely, it is outmost obligation, once in our research setting, to use our clinical knowledge to identify areas of needs. We must develop new devices to help our future patients even better than our current ones. Now that programs exist to acquire the *skillset*, it is up to us to change the *mindset*. Because we cannot fall asleep on our predecessor’s laurels.

## Competing interests

Dan Azagury is part of the Faculty of the Stanford University Biodesign program.
